# Prediction and Numerical Study of Thermal Performance of Gradient Porous Structures Based on Voronoi Tessellation Design

**DOI:** 10.3390/ma15228046

**Published:** 2022-11-14

**Authors:** Xiang Zhang, Minghao Zhang, Chenping Zhang, Tian Zhou, Xuncheng Wu, Xuezheng Yue

**Affiliations:** 1 School of Materials Science and Engineering, University of Shanghai for Science and Technology, No. 516, Jungong Road, Shanghai 200082, China; 2 National Center for Stomatology, National Clinical Research Center for Oral Diseases, Shanghai Key Laboratory of Stomatology, Department of Oral Maxillofacial-Head and Neck Oncology, Shanghai Ninth People’s Hospital, Shanghai Jiao Tong University School of Medicine, College of Stomatology, Shanghai Jiao Tong University, Shanghai 200011, China; 3 Yancheng Institute of Technology, Yancheng 224000, China

**Keywords:** porous structure, Voronoi, effective thermal conductivity, finite element analysis, prediction calculation

## Abstract

Porous materials are a new type of engineering material with both functional and structural properties. Compared with regular porous structures and random porous structures, a gradient porous structure is a porous structure with a spatial variation mechanism, which can adjust the layout of the structure by changing its own load and boundary conditions according to different situations, thus obtaining better performance. In this paper, three spatial Voronoi structures with different spatial gradients are designed using the spatial Voronoi tessellation method. The differences in thermal protection performances between the Voronoi spatial gradient structure and the regular structure and the effects of porosity, gradient direction and heat flow density on the three-dimensional Voronoi stochastic gradient structure were investigated via data simulation. The results show that the effective thermal conductivity of the Voronoi spatial gradient structure is lower than that of the regular structure. The effective thermal conductivity of the structure gradually decreases with increasing porosity. Taking the gradient Voronoi structure consisting of 3 × 3 × 3 units as an example, when the porosity increases from 83% to 94.98%, its effective thermal conductivity decreases from 0.586 to 0.149 Wm${}^{-1}$K${}^{-1}$. The anisotropy of the random structure leads to effective thermal conductivity errors of more than 5% in all three gradient directions. In addition, according to the principle of thermal resistance superposition, we designed a battery pack set for calculating the effective thermal conductivities of pillar-based porous materials, including three-dimensional Voronoi gradient random porous materials on the Grasshopper platform. In this way, the effective thermal conductivity of a pillar-based porous material can be predicted more accurately. The predicted calculation results and the simulation results basically agree with each other, and the relative errors of both are within 10%.

## 1. Introduction

Porous structures are characterized by a low relative density, a large specific surface area, high specific mechanical properties, and high designability [1,2], which can well meet the performance requirements of thermal protection integrated structures [3]. Compared with the regular porous structure and the random porous structure, the gradient porous structure is a porous structure with a spatial variation mechanism; it can adjust the layout of the structure by changing its own load and boundary conditions according to different situations, thus obtaining better performance [4]. Ali et al. [5] investigated the effect of a gradient porous structure on the melting behavior of phase change materials. Compared with the regular porous structure, the gradient porous structure can effectively improve the thermal performance of the energy storage system and equip the whole structure with a more uniform heat transfer distribution. Mo et al. [6] investigated the boiling heat transfer performance of porous structures with radial diameter gradients. Muzaki et al. [7] used numerical simulations to study the properties of gradient materials in battery applications. The results show that the heat transfer performance of the gradient porous structure is obviously better than that of the uniform porous structure. These studies show that gradient porous structures have excellent potential for thermal performance.

The Voronoi tessellation technique is defined as a random distribution of multiple seed points in a space. For each seed point, a vertical bisector is made with its neighboring points, and a closed space body from these vertical bisectors is then determined [8,9,10]. The Voronoi tessellation technique is often used in the structural design of porous materials such as foam metal materials, and cancellous and cortical bone in human bones [11,12,13]. Zhang et al. [14] designed different structures by varying the number of seed points and investigated the effect of the number of seed points and other structural parameters on the effective thermal conductivity of the porous structures. Li et al. [15] explored the radiative properties of Voronoi open-hole structures for the thermal applications of such structures. Du et al. [16] proposed a new approach based on porous structures of Voronoi tessellation with structures that can better meet the requirements of artificial bone implantation. The Voronoi porous materials have promising applications in energy absorption devices, human implants [17], and heat sinks [18] due to their controllable parameters and excellent mechanical and thermal properties.

With the development of diversified and complex porous structures, the requirements for material-forming technologies are becoming higher and higher [19,20]. Additive manufacturing (AM), also known as 3D printing, rapid prototyping, and material accumulation manufacturing [21], is a technology for manufacturing parts based on 3D model data via the “bottom–up” layer-by-layer stacking of materials [22,23,24]. This feature allows additive manufacturing technology to accurately manufacture any complex shape or personalized parts based on 3D digital models, and it does not require traditional tools and molds, which greatly reduces the process flow, realizes the rapid free-form manufacturing of 3D entities, and solves the manufacturing difficulties caused by the complex geometric features of porous materials [22]. In aerospace, AM porous structures often exhibit better properties in terms of mechanical performance and energy absorption [25,26,27]. In the biomedical field, AM porous structures can be applied to personalize custom bone implants by adjusting the elastic modulus to reduce stress shielding to meet the biocompatibility of the bone implant [28,29,30]. In the field of thermal protection, many scholars have interlinked AM porous structures with bionics to develop and design corresponding porous structures through the study of biological structures, realizing the integrated bionics-design-application research, and revealing the excellent properties of porous structures in terms of thermal performance [4,31,32]. Almonti et al. [33] fabricated metal foam structure using AM and studied the influence of pores per inch, branch thickness, and edge morphology of the structure on the effective thermal conductivity of the overall structure.

At present, the research on porous structures for additive manufacturing mainly focuses on the influence of structural parameters on the mechanical properties of materials such as elastic modulus, compressive strength [16,34,35] and energy absorption [36,37], while the research on the thermal conductivity of porous structures is not comprehensive. Additionally, most of the porous materials studied are homogeneous porous materials. However, in engineering applications, the loads on porous materials are often very complex, and homogeneous porous materials are usually unable to meet the application requirements. Especially in aerospace applications, porous materials should not only meet the requirements of lightweight but also have excellent properties such as thermal insulation [38,39,40]. Therefore, how to balance the relationship between mechanics and thermodynamics in materials has been the focus of numerous scholars [41,42,43].

In thermal protection systems, many structures exhibit excellent thermal insulation performance, especially in gradient structures. Lin et al. [3] designed regular structures that had different gradients of thermal protection and selected the gradient structure with the best thermal insulation performance from the four regular gradient structures by the finite element method. However, the conventional gradient structures are characterized by simple heat transfer paths and homogeneous designs, which hinder their diverse applications in thermal protection. In this paper, the gradient design is used in combination with the Voronoi random structure, which is compared with the previous design of the Voronoi random structure as shown in Table 1. A Voronoi random porous structure design method with different spatial gradients is proposed based on the Voronoi Mosaic design. We designed three Voronoi random porous structures with different gradients, and three corresponding regular structures, respectively, and controlled the porosity by changing the pillar radius. Then, the finite element analysis method is used to calculate the thermal behavior of the random gradient structure and the regular structure, respectively. In addition, based on the superposition principle of thermal resistance, a method to predict the effective thermal conductivities of three-dimensional Voronoi random porous materials is proposed.

## 2. Materials and Methods

### 2.1. Design of the Porous Structure

The design method of the porous structure is based on Voronoi tessellation. The Voronoi structure was first proposed by the Russian mathematician Voronoi and applied to three-dimensional space [12]. Voronoi tessellation as a spatial partitioning method is characterized by a closed space with many discrete seed points, with each seed point as the center of a circle expanding outward at a uniform rate, and stopping the expansion when the boundaries of two circles touch and form a new boundary. The mathematical model of Voronoi space partitioning is defined as [16,44]:

(1) $$C_{i}=\left\{O|d\left(O,S_{i}\right)\leq d\left(O,S_{j}\right),\forall j\neq i,j=1,\ldots ,n\right\}$$

where

1. $C_{i}$ is the Voronoi polyhedral space cell corresponding to the seed point $S_{i}$;
2. $d(O,S_{i})$ is the Euclidean distance between the location point O and the seed point $S_{i}$;
3. $S_{i},\ldots ,S_{n}$ is a series of seed points defined in Euclidean space.

**Figure 1:**
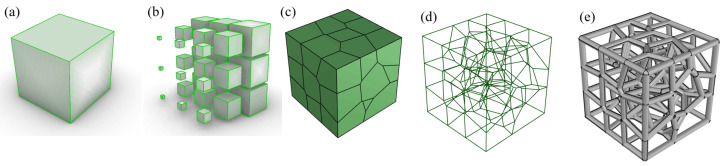
Design process of gradient Voronoi structure: (**a**) Select the cube as the design space. (**b**) Divide the square into nine small squares with equal differences. (**c**) Randomly generate a seed point inside each small cube and Voronoi polyhedrons with common edges. (**d**) Frame lines of gradient Voronoi structure. (**e**) Gradient Voronoi.

**Figure 2:**
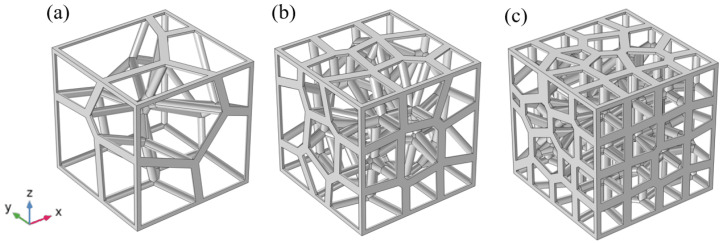
Strut-based gradient Voronoi structures: (**a**) Gradient Voronoi structure consisting of 2 × 2 × 2 units (GV2). (**b**) Gradient Voronoi structure consisting of 3 × 3 × 3 units (GV3). (**c**) Gradient Voronoi structure consisting of 4 × 4 × 4 units (GV4).

In this study, the porous structure was designed by using the software Rhinoceros 7 (McNeal, Seattle, WA, USA) with the parametric design plugin Grasshopper (v.1.0.0007). The design process of the gradient porous structure based on the Voronoi mosaic is shown in Figure 1. First, the sample space is divided into nine identical small cubes on average. Sencond, these cubes are divided into gradients in arithmetic progression, creating a random point for each small cubic space; these random points are also called seed points. This design allows each seed point to be distributed as a spatial gradient in the sample space. Third, based on the location of these seed points, a 3D Voronoi diagram can be generated directly in Grasshopper. Fourth, we extract the borderline of the 3D Voronoi diagramis extracted. Fifth, a porous structure with the pillar radius D for the borderline of the 3D Voronoi diagram is created. Based on the same design method, we only changed the number of elements to design three porous structures with different gradients, as shown in Figure 2.

The thermal protection system in aerospace should not only meet the requirements of lightweight but also have heat insulation performance. Therefore, we designed five different high porosities from 80% to 95% for three different gradients of the Voronoi structure, and the porosity of the porous structure was defined as [14]:

(2) $$P=1-\varphi =1-\frac{V_{cs}}{V_{m}}$$

where *P* is the porosity of the porous material, φ is the volume fraction of the porous material, $V_{m}$ is the volume of the sample as a whole, and $V_{cs}$ is the volume of the porous structure.

**Figure 3:**
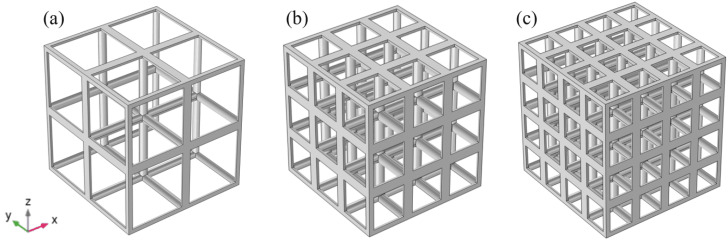
Regular porous structure: (**a**) Regular porous structure consisting of 2 × 2 × 2 units (RP2). (**b**) Regular porous structure consisting of 3 × 3 × 3 units (RP3). (**c**) Regular porous structure consisting of 4 × 4 × 4 units (RP4).

In order to contrast the properties differences between the gradient and non-gradient structures, we designed three regular structures corresponding to the three different gradient structures, as shown in Figure 3. This regular structure is formed by stacking simple cubic singletons. The simple cubic structure excels in yield strength and elastic modulus [45,46], but its difference from this gradient random structure is not yet known. The standard space for all of the above samples is 30 × 30 × 30 mm${}^{3}$. In the work, all of the structural parameters studied are shown in Table 2.

**Figure 4:**
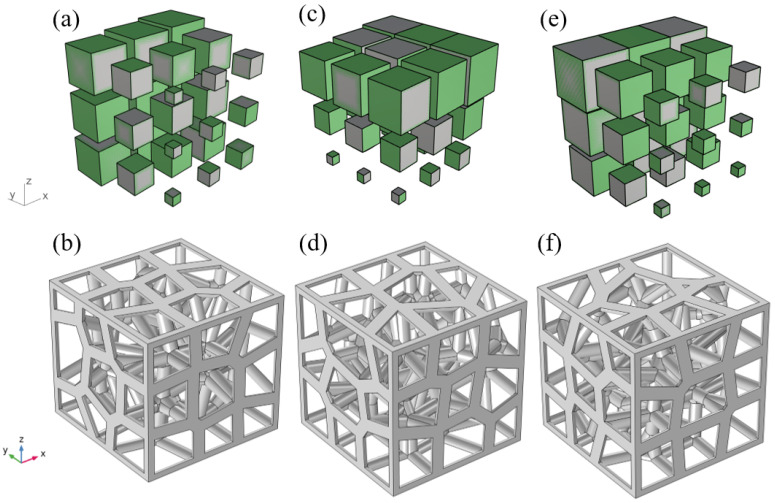
Gradient variation model of GV3 structure with 89.09 and porosity in OZ, OY, OX directions: (**a**) Gradient change in OZ direction; (**b**) OZ gradient structure; (**c**) Gradient change in OY direction; (**d**) OY gradient structure; (**e**) Gradient change in OX direction; (**f**) OX gradient structure.

In general, the random distribution of pores in a random porous material makes the influence of structural orientation on its properties very weak. For random gradient porous materials, seed points are generated depending on the location and size of the gradient range, which necessitates the consideration of whether different gradient orientations have an influence on the properties of porous materials. Therefore, to investigate the effect of gradient orientation on the properties of porous materials, the OX, OY, and OZ directions of the porous materials studied in this work are shown in Figure 4. Taking the GV3 structure as an example, the original OZ gradient structure is based on the seed point in the center as the standard point, and the OX direction along the equivariant series variation is shown in Figure 4a, which results in the gradient structure in the OZ direction, as shown in Figure 4b. Rotate the GV3 structure in the OZ direction by 90° counterclockwise along the X-axis, as shown in Figure 4c, to obtain the gradient structure in the OY direction, as shown in Figure 4d. Rotate the GV3 structure in the OZ direction 270° counterclockwise along the Y-axis, as shown in Figure 4e to obtain the gradient structure in the OX direction as shown in Figure 4f.

### 2.2. Finite Element Simulation of Heat Transfer

In order to investigate the effective thermal conductivity of gradient porous structures based on Voronoi mosaic design, the software COMSOL^®^ v. 5.4 (COMSOL Inc., Stockholm, Sweden) was applied to numerically simulate the porous structures in the steady state and transient state. The specific steps include the definition of material properties, the imposition of loads and boundary conditions, the delineation of the mesh, and the calculation of the results.

#### 2.2.1. Governing Equations and Boundary Conditions

The heat transfer behaviors of porous structures are very complex. On the one hand, the low temperatures set in this study and the small size of the cells of the structure will result in a very slow flow of fluid in the pores. On the other hand, the thermal conductivity of the solid phase metal is much higher than that of the fluid. Therefore, thermal radiation and convective heat transfer from the structure are not considered. The controlled heat transfer equation can be expressed as:

(3) $$\rho C_{p}\nabla T+\nabla \left(-\lambda \nabla T\right)=Q$$

where ρ is the initial density of the material, $C_{p}$ is the initial specific heat capacity, *T* is the temperature, λ is the initial material thermal conductivity, and *Q* is the total heat.

**Figure 5:**
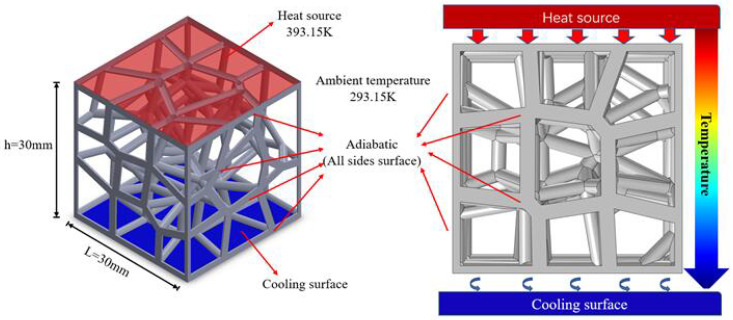
The temperature loading and boundary conditions of thermal simulation of GV3 structures.

The physical parameters corresponding to the Ti6Al4V applied in this study as the matrix material at different temperatures are shown in Table 3. Taking the GV3 structure as an example, its load and boundary conditions in the numerical simulation are shown in Figure 5: the upper surface of the structure is a constant heat source with a temperature of 393.15 K. The lower surface is the cooling surface, the ambient temperature is 293.15 K, and the rest of the surfaces are set to be thermally insulated.

#### 2.2.2. Mesh Independence Tests

**Figure 6:**
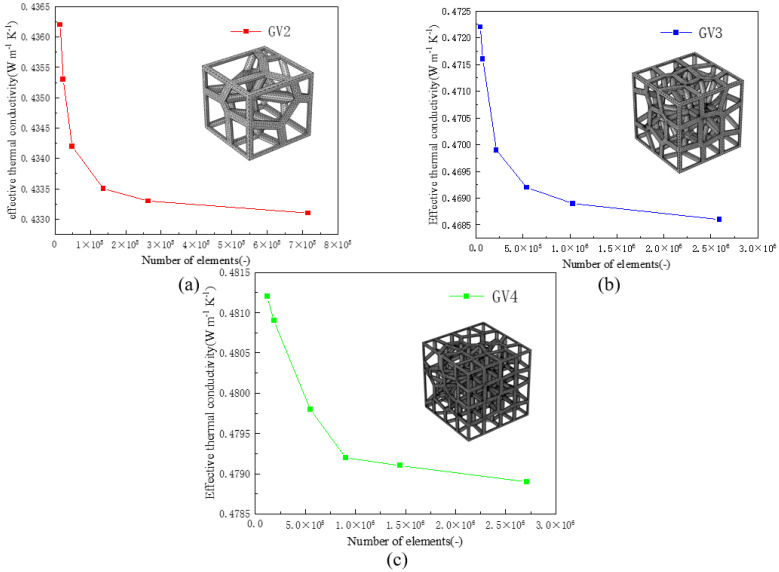
Results of mesh independence tests of three gradient structures with 86% porosity: (**a**) GV2; (**b**) GV3; (**c**) GV4.

The mesh independence test is a critical factor to validate the numerical simulations. The results of meshing different structures with different mesh sizes as shown in Table 4. In this process, mesh independence tests were performed, with three gradient random structures with 86% porosity, as shown in Figure 6, and six groups of grids were named as very rough, rough, general, fine hyperfine, and finest according to the mesh size, respectively. The results show that the effective thermal conductivity of all three structures decreases with the increase in the number of elements, and it eventually tends to be stable. The effective thermal conductivities of the fine mesh are 0.4335 Wm${}^{-1}$K${}^{-1}$, 0.4692 Wm${}^{-1}$K${}^{-1}$, and 0.4794 Wm${}^{-1}$K${}^{-1}$, respectively, which are 4.15%, 6.39%, and 3.75% lower than those of the very rough mesh. Respectively, it is 0.09%, 0.13% and 0.13%, higher than the effective thermal conductivities corresponding to finest mesh. Considering the calculation time and the accuracy of the calculation results, the mesh size corresponding to the fine mesh as the optimal mesh size was chosen for the finite element simulation.

#### 2.2.3. Prediction Calculation

Thermal resistance analysis is a well-established method for calculating the effective thermal conductivities of porous materials in complex heat transfer processes. As mentioned earlier, when the convective and radiative heat transfer processes are not considered and the thermal conductivity of a solid far exceeds that of any other state, the heat conduction in the solid phase skeleton dominates the thermal conductivity process of its overall structure. Therefore, the total thermal resistance of the porous material can be calculated by introducing the concept of thermal resistance based on the spatial arrangement of the solid-phase skeleton. Thus, the thermal conductivity of the porous structure is obtained. The spatial arrangement of the three-dimensional Voronoi gradient random structure is irregular, and it is very tedious to calculate manually. Therefore, a battery pack was constructed on the Grasshopper platform that can calculate the effective thermal conductivities of pillar-based porous structures, including three-dimensional Voronoi gradient random porous materials, to provide some theoretical support for the design of porous materials for thermal control applications.

In this paper, the temperature gradient direction of the structure as a whole is parallel to the vertical direction. Therefore, to simplify the calculation, the contribution of the struts on the horizontal plane perpendicular to the direction of the temperature gradient to the overall effective thermal conductivity of the structure is neglected. Taking the Voronoi porous structure as an example, the four prisms of the porous structure, the outer frame and the line segments corresponding to the inner structure are first filtrated. The nodes are then layered, the length l of the line segments within each layer cell is calculated, the number *N*, the thermal conductivity of the pillar, is considered as a one-dimensional thermal conductivity problem along its length direction, and the thermal resistance within each layer cell is a parallel superposition, which can be expressed as:

(4) $$R_{x}=\frac{1}{\sum_{j=1}^{N}\frac{\lambda_{s}\pi r^{2}}{bl_{xj}}}$$

where $R_{x}$ is the total thermal resistance of the $x^{th}$ layer cell, *N* is the total number of line segments in the $x^{th}$ layer cell, $l_{xj}$ is the length of the $j^{th}$ line segment in the $x^{th}$ layer cell, the value of *b* depends on the spatial location where the line segment is located, and for the structure of the four prongs of the structure, the outer frame and the inner line segments, the corresponding *b* values are 4, 2, and 1, respectively.

The thermal resistance between the layer units is superimposed in series so that the total thermal resistance of the structure can be expressed as:

(5) $$R=\sum_{x=1}^{G}R_{x}$$

where *G* is the number of layer cells.

The thermal resistance, as the resistance of the heat transfer process, can be expressed as the quotient of the power of the heat transfer process and the amount transferred in the process: that is, the quotient of the temperature difference and the heat flow rate. Thus, based on the above definition and Fourier’s law, as shown in Equations (4) and (5), an expression for the effective thermal conductivity of the pillar-based porous structure can be obtained as follows:

(6) $$\lambda_{\mathrm{eff}\;}=\frac{L}{A_{s}\sum_{x=1}^{G}R_{x}}$$

**Figure 7:**
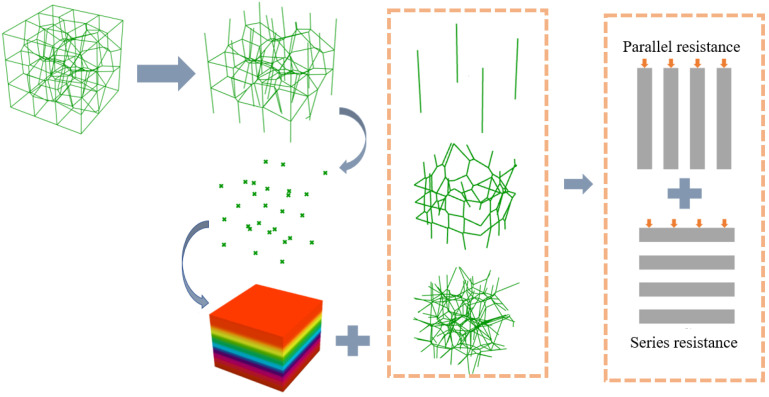
Calculation of the effective thermal conductivity of three-dimensional Voronoi random porous materials.

**Figure 8:**
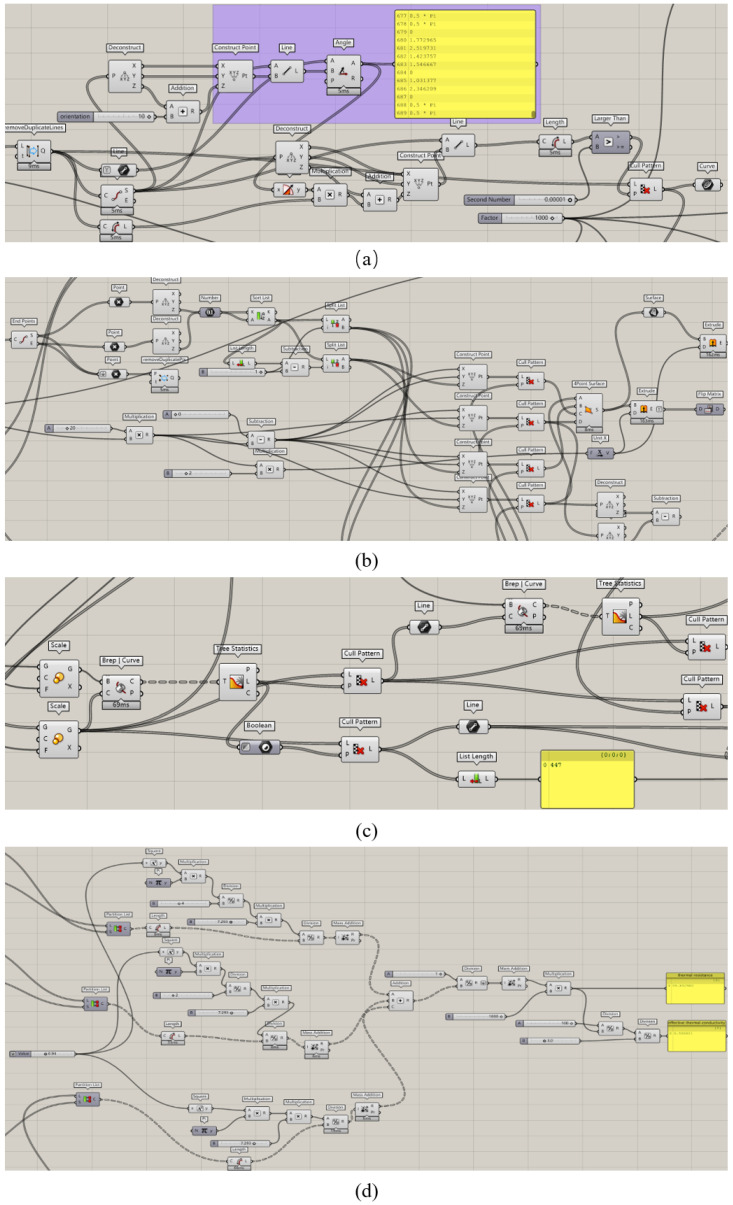
Cell logic diagram for calculating the effective thermal conductivity of a pillar-based porous structure on the Grasshopper platform: (**a**) line segment screening module; (**b**) layer cell division module; (**c**) separation module of the four prongs, outer frame and inner line segments of the structure; (**d**) thermal resistance calculation.

The calculation of the effective thermal conductivity of the three-dimensional Voronoi random porous material is shown in Figure 7. The cell logic diagram for calculating the effective thermal conductivity of the pillar-based porous structure on the Grasshopper platform is shown in Figure 8.

## 3. Results

The effective thermal conductivity of the three-dimensional Voronoi gradient random porous material with different porosities, gradient structures and orientations obtained from the finite element simulation at steady state is shown in Table 5.

### 3.1. Verification of the Accuracy of the Simulation Results

**Figure 9:**
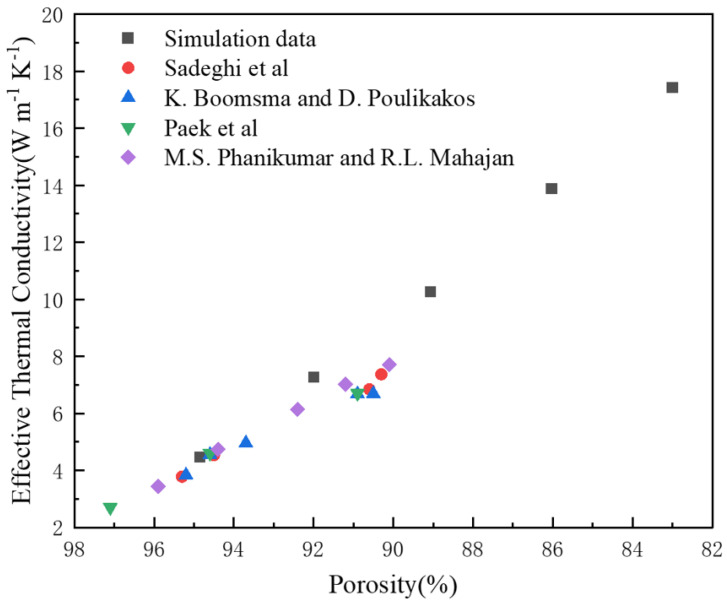
Comparison of simulation data and experimental data in the literature [47,48,49,50].

To verify the accuracy of the finite element simulation, aluminum 6101 was used as the base material, and the method described in Section 2.2 was used for modeling. The physical properties of 6061 aluminum alloy are as follows: a thermal conductivity of 218 Wm${}^{-1}$K${}^{-1}$, a density of 2700 kg/m${}^{3}$, and specific heat capacity of 963 J/kg K. Effective thermal conductivity of 6061 aluminum alloy GV3 structure with different porosity as shown in Table 6. Figure 9 shows the comparison between the effective thermal conductivity of the simulated RV3 structure with different porosities and the results of the experimental data of Sadegji et al. [47], K. Boomsma and D. Poulikakos [48], Paek et al. [49], and M.S. Phanikumar and R.L. Mahajan [50]. With a porosity of 92% or more, the present simulated data of the work are in general agreement with the experimental data in the literature. However, at a porosity of below 92%, the simulated data deviate from the experimental data in the literature, but the errors are less than 10%. This difference may be caused by the simplification of the finite element model, the heat loss in the experiment, and the influence of contact thermal resistance on the measurement results. In summary, the simulation data results are basically consistent with the comparison of the literature experimental data results, and the finite element model construction method is correct. The simulation results are basically accurate.

### 3.2. Effect of the Gradient Structure on the Effective Thermal Conductivity

**Figure 10:**
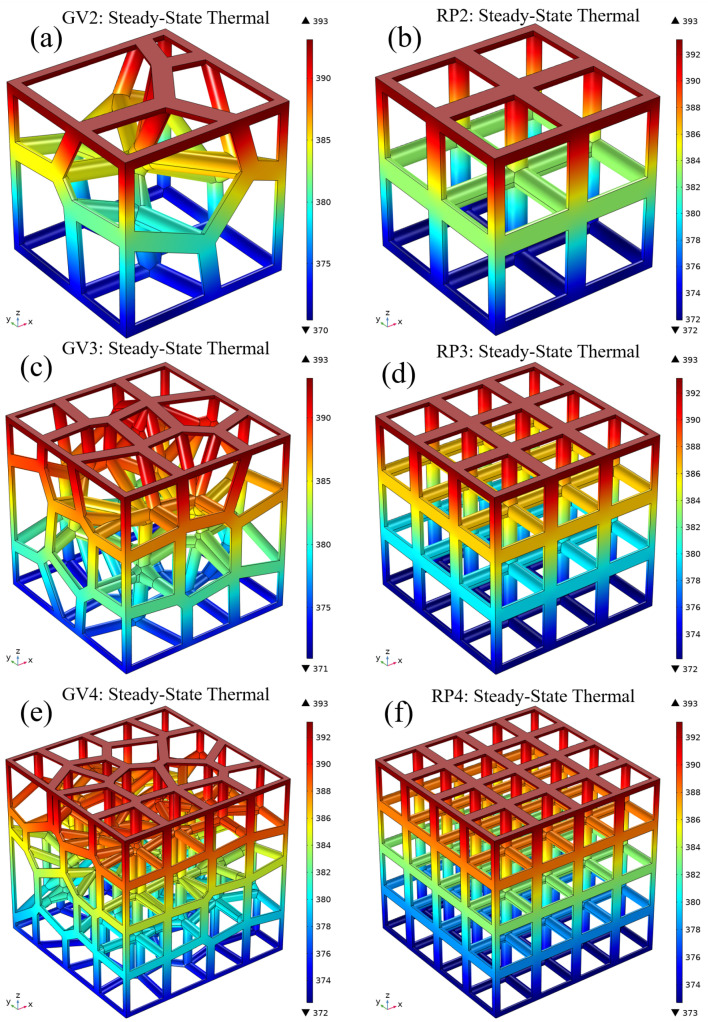
The steady-state temperature distributions for six structures with 89% porosity: (**a**) GV2; (**b**) GV3; (**c**) GV4; (**d**) RP2; (**e**) RP3 (**f**) RP4.

Figure 10 shows the steady-state temperature distributions of the six structures with 89% porosity. The upper surface of all structures is constant, at 393.15 K. The different structures show different temperature distributions under the same load and boundary conditions. In the temperature distribution diagram of the gradient-free structure, the heat is transferred from the top surface to the bottom surface, and the temperature decreases with the increase in the transfer distance. The uniform distribution of nodes in the gradient-free structure leads to a more uniform temperature distribution. Among the three gradient-free structures, the RP2 structure has the smallest number of nodes, which makes the heat dispersion in the structure the smallest, so that the RP2 structure has the lowest temperature on the lower surface (371.73 K). In the gradient structure, the gradient distribution of the seed points leads to the formation of nodes that are also gradient distributed, making the temperature distribution of the gradient structure uneven, and thus affecting the effective thermal conductivity of the overall structure. In addition, it is obvious in the temperature comparison between the gradient structure and the gradient-free structure that the lower surface temperature of the gradient structure is generally lower than the lower surface temperature of the gradient-free structure.

**Figure 11:**
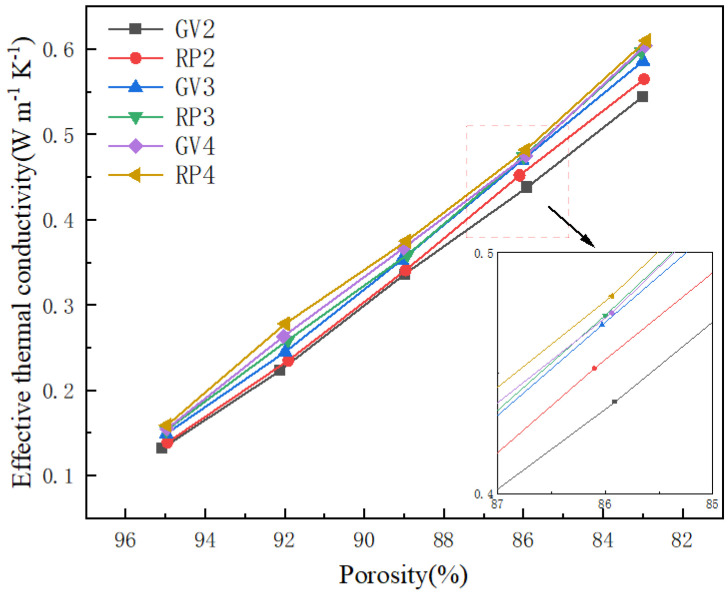
Curve of effective thermal conductivity with porosity for different structures.

In order to further investigate the effect of the gradient structure on the effective thermal conductivity, five porosities of six different structures were selected to perform steady-state thermal simulation experiments, and the variation curve of the effective thermal conductivity with porosity is shown in Figure 11. The results show that the effective thermal conductivities of both three-dimensional Voronoi gradient random porous materials and regular materials increases with decreasing porosity. Taking the GV3 structure as an example, when the porosity decreases from 94.98% to 83%, its effective thermal conductivity increases from 0.149 to 0.586 Wm${}^{-1}$K${}^{-1}$, and the two are negatively correlated. The radius of the struts is a key parameter in controlling the porosities of three-dimensional Voronoi gradient random porous materials. The larger the radius of the struts, the larger the volume fraction of the solid phase skeleton, the smaller the porosity, and the smaller the volume fraction of air in the fluid phase. Since the thermal conductivity of the solid-phase skeleton is greater than that of air, the effective thermal conductivity of the structure increases with decreasing porosity. More interestingly, the effective thermal conductivity of the regular structure is always higher than that of the gradient random structure for an equal number of unit cells. The nodes generated by the gradient random structure are an important factor affecting the effective thermal conductivity of the three-dimensional Voronoi gradient random porous material. Compared with the regular structure, the nodes of the gradient random structure are connected to the pillars in a more complex manner, which also leads to more severe heat loss at the nodes of the gradient random structure, and so the effective thermal conductivity of the regular structure is always higher than the effective thermal conductivity of the gradient random structure. At the same porosity, the RP4 structure has the highest effective thermal conductivity among the six structures, which is followed by the GV4 structure, RP3 structure, GV3 structure, RP2 structure, and GV2 structures.

### 3.3. Effect of the Gradient Direction on the Effective Thermal Conductivity

**Figure 12:**
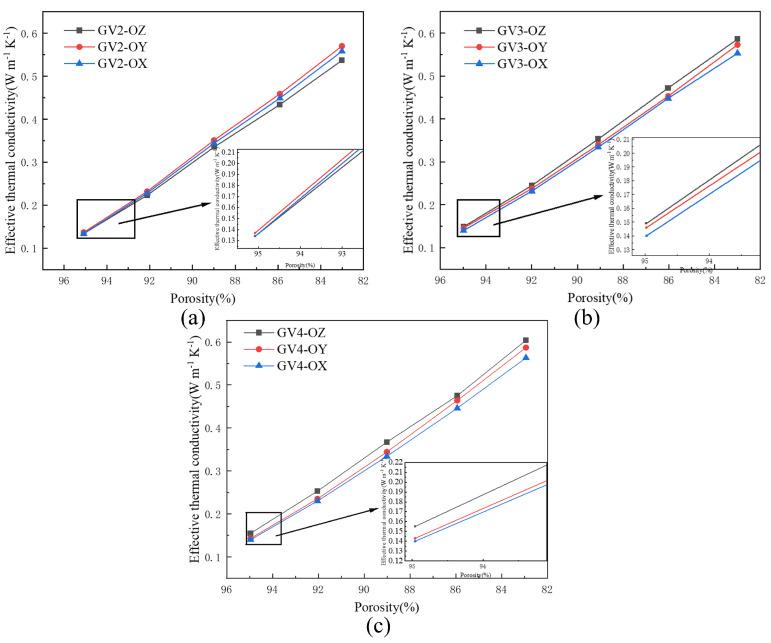
Effective thermal conductivity of various voronoi porous structures with different gradient orientations: (**a**) GV2; (**b**) GV3; (**c**) GV4.

To investigate whether the three-dimensional Voronoi gradient random structures are anisotropic, the corresponding effective thermal conductivities of the GV structures with different porosities were plotted in the OX, OY, and OZ directions in Figure 12a–c. The result shows that the difference in the effective thermal conductivities in the direction of the three GV structures increases as the porosity decreases. At 95% porosity, the maximum difference in the effective thermal conductivity in each direction does not exceed 0.015 Wm${}^{-1}$K${}^{-1}$, while at 83% porosity, the maximum difference in the effective thermal conductivity in each direction reaches 0.041 Wm${}^{-1}$K${}^{-1}$. For the GV2 structure, the minimum difference in effective thermal conductivity due to different orientations was 2.19%, and the maximum difference was 5.79%; the minimum difference in effective thermal conductivity due to different orientations was 5.08%, and the maximum difference was 6.04% for the GV3 structure; while the minimum difference in effective thermal conductivity due to different orientations was 6.10%, and the maximum difference was 9.68% for the GV4 structure. Since the difference in effective thermal conductivity of all three structures in the direction exceeds 5%, it can be assumed that the three-dimensional Voronoi gradient random structure has anisotropic thermal conductivity. More interestingly, the effective thermal conductivity in the OY direction of the GV2 structure is significantly higher than those in the OZ and OX directions; however, this situation changes in the GV3 and GV4 structures, where the OZ direction is higher than the effective thermal conductivity in the other directions. Therefore, it is more convincing that the thermal conductivity of the three-dimensional Voronoi gradient random structure is anisotropic.

### 3.4. Heat Flux Density Distribution

**Figure 13:**
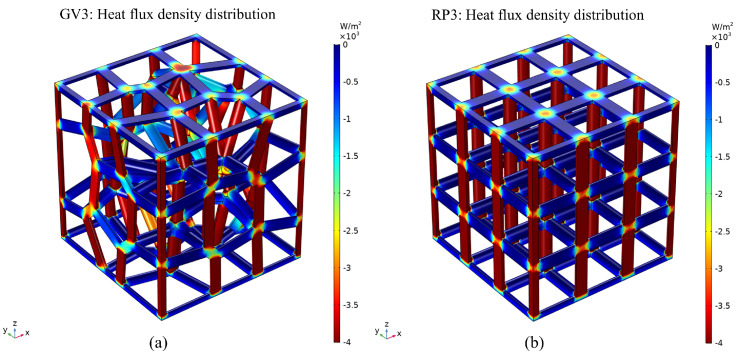
Heat flux density distribution of porous materials of different structural types with 89% porosity: (**a**) GV3; (**b**) RP3.

The heat flux density distribution of the GV3 structure and the RP3 structure with 89% porosity is shown in Figure 13. The higher heat flux density means that this part of the region captures the thermal conductivity of the substrate material more effectively, which contributes more to heat transfer for a given temperature load. Figure 13 shows that the regular structure has a more uniform heat flux density distribution compared to the three-dimensional Voronoi gradient random porous material. This is because the regular structure is a porous material consisting of its individual structural units stacked in a uniform arrangement in space, and the magnitude of the heat flux density is uniformly distributed for the overall structure. Figure 12a,b show that the smaller heat flux density in the structures occurs mainly in the planar region perpendicular to the direction of the temperature gradient in both structures. The pillars in the three-dimensional Voronoi gradient random porous material are randomly distributed in space and the magnitudes of their heat flux density are no longer uniformly distributed; in addition, heat is mainly transferred along the pillars that are parallel to or at an angle to the direction of the temperature gradient. In addition, maximum heat flux densities of 4000 w/m for the GV3 structure and 3500 for the RP3 structure occur in pillars with the same OZ direction as the temperature gradient.

### 3.5. Prediction of Effective Thermal Conductivity of Porous Structures

As can be seen from Table 7, the relative errors between the simulated and predicted results range from 0% to 10%, with the smallest error of 0% for the GV2 structure, with 95.08% porosity, and the largest error of 8.77% for the GV4 structure, with 82.94% porosity. The prediction results are all smaller than the simulation results, and the relative errors of both increase with decreasing porosity.

## 4. Discussion

The gradient structure can influence the various properties of porous structures. Therefore, the gradient structure for three-dimensional Voronoi random porous materials has also received much attention. As mentioned in Section 3.2, the effective thermal conductivity of the three-dimensional Voronoi gradient random structure is significantly lower than that of the regular structure without gradient. This is because the range of seed points in the three-dimensional Voronoi varies in gradient, resulting in the randomness of each seed point also varying. However, due to the gradient range constraint, the seed point in each gradient range always keeps a certain distance from the seed point in another gradient range. Therefore, when forming the gradient structure, the pillars between the seed points and the seed points will remain tilted, which is a dramatic change from the vertical or horizontal state of the regular structure pillars. In Section 3.4, it is the difference in the morphology of the two structural pillars that leads to their heat flux density distribution difference. The heat will be transferred along the direction of the temperature gradient in the struts. Therefore, the shorter the length of the pillar in the direction of the temperature gradient, the faster the heat transfer will be. To verify the accuracy of the simulation results, five models with different porosities were designed for each structure. As shown in Figure 11, the change in porosity does not affect the result where the effective thermal conductivity of the gradient structure is lower than that of the gradient-free structure. It can be shown that the introduction of the gradient structure can reduce the effective thermal conductivity of the porous material to some extent. Meanwhile, porosity is an important parameter that affects the performances of porous structures. The size of the porosity is determined by the size of the pillar radius of the pillar-based porous structure. As the pillar radius of the porous structure decreases, the porosity of the structure increases and the rate of heat transfer decreases, ultimately leading to the higher porosity of the porous structure and the lower effective thermal conductivity. This is also reflected in Figure 11, where the effective thermal conductivity of the various structures decreases substantially as the porosity increases from 83% to 95%, which also provides thermal protection to some extent. However, the higher porosity also means that the pillars of the porous structures are more fragile, which can affect the mechanical properties of the overall structure. Therefore, balancing the mechanical and thermal properties of porous structures is an issue of attention.

The anisotropy of the porous structures is also a key concern for many researchers. As mentioned in Section 3.3, the maximum effective thermal conductivities of the GV3 and GV4 structures appears in the OZ direction, and the minimum effective thermal conductivity appears in the OX direction. Furthermore, the maximum thermal conductivity of the GV2 structure appears in the OY direction, and the minimum effective thermal conductivity appears in the OZ direction. Only in the GV2 structure with 95% porosity can the difference in effective thermal conductivity in the three directions be less than 5%. The differences in the effective thermal conductivities of the remaining structures were all above 5%. This discrepancy is mainly due to the randomness of the three-dimensional Voronoi gradient stochastic structure. The distribution of the pillars in different directions is different, and the path of heat transfer changes dramatically. At the same time, the difference of the effective thermal conductivity of the porous structures increases with the increase in porosity. This indicates that the three-dimensional Voronoi gradient random structure is anisotropic in terms of thermal conductivity. Therefore, orientation is also a key factor affecting the thermal conductivity of the porous structures. The prediction of the effective thermal conductivity of porous materials enables researchers to obtain an approximate understanding of the thermal properties of a structure at the design stage. In Section 3.5, the predicted results of the three Voronoi gradient stochastic structures were compared with the simulated results, and the predicted results were wholly found to be smaller than the simulated results. On the one hand, this results in the fact that the prediction method treats all the cross-sectional areas of the pillars as circles of the same radius when calculating, which brings about a calculated value of the cross-sectional area of the column near the node that is greater than the actual modeled value. The thermal resistance near the nodes is increased, so that the predicted values of the effective thermal conductivity of the structure are smaller than the results of the simulation. On the other hand, as the radius of the pillar increases, the porosity of the structure decreases, and the repeatedly calculated cross-sectional area of the pillar near the node is then increased. With the gradual underestimation of the effective thermal conductivity obtained from the prediction, its relative error with the simulation results increases. It is worth pointing out that the greater the number of the unit cell is, the greater the number of nodes generated. The number of line segments in the structure also increases significantly, which greatly increases the error value of the prediction process. The Grasshopper platform is not a professional calculation software, so the prediction results may be subject to errors due to the written battery logic program and the instability of the software. Despite the above problems, the predicted results are in general agreement with the simulated results, and the relative errors of both are within 10%. It is shown that the prediction method can quickly and more accurately predict the effective thermal conductivities of pillar-based porous structures at the design stage of porous materials with high porosity, providing a certain reference for the design of porous materials used in thermal control applications.

## 5. Conclusions

In the work, we used the Voronoi tessellation-based spatial partitioning method to obtain three different gradient Voronoi random structures for the application of a thermal protection system. The thermal conductivity of the three-dimensional Voronoi gradient random structure was also studied in the steady state, and the effects of gradient structure, orientation and porosity on its effective thermal conductivity were analyzed. The following conclusions can be drawn from this study:

1. The effective thermal conductivity of the three-dimensional Voronoi gradient random porous structure is smaller than that of the regular structure without a gradient for the same porosity. With the increase in porosity, the effective thermal conductivity of both structures decreases gradually. Therefore, the introduction of the gradient structure can effectively improve the thermal protection performance of the porous structure.
2. The effective thermal conductivity of the three-dimensional Voronoi gradient random porous structure is influenced by the orientation. In the case of the GV4 structure, for example, the difference in orientation leads to a maximum error of 9.68% in the effective thermal conductivity of the structure. Meanwhile, the errors in the effective thermal conductivity due to the different orientations of the three gradient structures almost all exceed 5%. Therefore, there is anisotropy in the thermal conductivity of the three-dimensional Voronoi gradient random structure.
3. The heat flux density represents the ability to transfer heat in a structure. The heat flux density distribution of the regular structure is more uniform than that of the three-dimensional Voronoi gradient random porous structure. This is because regular structures have fewer heat transfer paths. In contrast, the heat transfer path in the three-dimensional Voronoi gradient random porous structure is more complex and tortuous, increasing the thermal resistance and weakening the thermal conductivity of the material.
4. Based on the Grasshopper platform, a battery pack set was designed for calculating the effective thermal conductivity of pillar-based porous materials, including three-dimensional Voronoi gradient random porous materials, which allows for a more accurate prediction of the effective thermal conductivity of a pillar-based porous material. The predicted calculation results and the simulation results basically agree with each other, and the relative errors of both are within 10%. The cell set verifies the accuracy of the simulation results to a certain extent, and it also provides some reference for application to the design of thermal protection structures.

## Figures and Tables

**Table 1:** Comparison of design innovation points of Voronoi structure.

Model	Research Method	Innovation Points	Application	Cite
Voronoi structure	Finite element analysis	The Voronoi structures were generated by adjusting the number of seed points	Thermal conduction	[14]
Voronoi structure	Theoretical calculation	The Voronoi structures were generated by adjusting the foam porosity and pores per inch parameters	Radiative characteristics	[15]
Voronoi structure	Experiment	The Voronoi structures were generated by adjusting the three structural design parameters (strut diameter D, unit distance d, irregularity i)	Mechanical	[16]
Voronoi structure	Experiment	The Voronoi structures were generated by adjusting the three structural design parameters (pores per inch (PPI), branch thickness (r), and edges morphology (smooth-regular))	Convective heat transfer	[33]
Voronoi structure	Finite element analysis	The Voronoi structures were generated by adjusting the spatial gradient range of random points	Thermal conduction	-

**Table 2:** Specific geometric parameters of the porous structure.

Structures	Porosity (%)	Strut Radius (mm)	Volume of the Cellular Structures (mm${}^{3}$)	Surface Area (mm${}^{2}$)
	95.08	1.02	1327.90	3820.10
	92.12	1.31	2125.00	4721.90
GV2	88.99	1.59	2972.70	5512.80
	85.91	1.80	3803.70	6055.00
	83.01	1.99	4587.30	6507.70
	94.98	0.66	1355.40	5215.60
	91.99	0.84	2162.30	6298.40
GV3	89.09	1.00	2953.40	7147.00
	86.03	1.15	3771.40	7849.40
	83.00	1.29	4591.00	8432.40
	94.96	0.48	1360.80	6610.50
	92.04	0.61	2147.90	7973.50
GV4	89.01	0.73	2967.80	9055.50
	85.94	0.84	3797.00	9909.80
	82.94	0.94	4696.30	10,620.00
	94.95	1.17	1363.50	3827.70
	91.91	1.50	2184.30	4725.50
RP2	88.96	1.78	2980.80	5376.60
	86.10	1.98	3753.00	5888.70
	82.97	2.28	4598.10	6529.70
	94.98	0.78	1355.40	5019.20
	91.92	1.00	2181.60	6115.90
RP3	88.92	1.17	2991.20	6930.90
	86.00	1.33	3780.20	7638.20
	83.04	1.48	4579.80	8248.10
	94.96	0.58	1360.80	6074.70
	91.99	0.77	2162.70	7699.10
RP4	88.94	0.85	2985.50	8252.10
	85.94	1.00	3795.20	9370.20
	82.92	1.12	4611.60	10,152.00

**Table 3:** Thermo-physical properties of Ti6Al4V alloy.

Temperature (K)	Density (kg/m${}^{3}$)	Thermal Conductivity (Wm${}^{-1}$K${}^{-1}$)	Specific Heat (J/(kg K))
293.15	4429.989	7.076	536.041
313.15	4428.525	7.147	545.973
333.15	4425.977	7.285	553.497
353.15	4423.419	7.441	560.723
373.15	4420.850	7.613	567.660
393.15	4418.271	7.800	574.316

**Table 4:** The results of meshing different structures with different mesh sizes.

Structure	Very Rough	Rough	General	Fine	Hyperfine	Finest
GV2	14,944	23,535	48,666	137,164	263,210	714,978
GV3	46,080	71,778	216,828	538,697	1,030,911	2,590,053
GV4	117,376	184,728	547,497	900,518	1,447,680	2,707,665

**Table 5:** Effective thermal conductivity of three-dimensional Voronoi gradient random porous materials with different porosity, gradient structure and orientation.

Structure	Porosity (%)	Effective Thermal Conductivity (Wm${}^{-1}$K${}^{-1}$)		
		OZ	OY	OX
	95.08	0.134	0.137	0.134
	92.12	0.223	0.232	0.228
GV2	88.99	0.335	0.351	0.344
	85.91	0.434	0.459	0.449
	83.01	0.537	0.570	0.558
	94.98	0.149	0.146	0.140
	91.99	0.245	0.238	0.232
GV3	89.06	0.353	0.340	0.334
	86.03	0.472	0.453	0.448
	83.00	0.586	0.573	0.553
	94.96	0.155	0.143	0.140
	92.04	0.253	0.235	0.230
GV4	89.01	0.367	0.344	0.334
	85.94	0.475	0.464	0.446
	82.94	0.604	0.587	0.563
	94.95	0.139		
	91.91	0.235		
RP2	88.96	0.341		
	86.10	0.452		
	82.97	0.565		
	94.98	0.153		
	91.92	0.259		
RP3	88.92	0.359		
	86.00	0.474		
	83.04	0.597		
	94.96	0.159		
	91.99	0.278		
RP4	88.94	0.370		
	85.94	0.479		
	82.92	0.610		

**Table 6:** Effective thermal conductivity of 6061 aluminum alloy GV3 structure with different porosity.

Porosity (%)	94.85	91.99	89.06	86.03	83.00
Effective Thermal conductivity (Wm${}^{-1}$K${}^{-1}$)	4.46	7.26	10.26	13.88	17.42

**Table 7:** Calculated results of predicted effective thermal conductivity of three-dimensional gradient random porous materials based on Voronoi division.

Structure	Porosity (%)	Predicted Results (Wm${}^{-1}$K${}^{-1}$)	Relative Error (%)
	95.08	0.134	−0.00
	92.12	0.221	−0.90
GV2	88.99	0.326	−2.98
	85.91	0.418	−4.57
	83.01	0.511	−6.07
	94.98	0.143	−0.67
	91.99	0.232	−1.22
GV3	89.09	0.329	−3.12
	86.03	0.434	−4.03
	83.00	0.547	−6.66
	94.96	0.145	−5.81
	92.04	0.234	−6.00
GV4	89.01	0.336	−7.63
	85.94	0.445	−8.42
	82.94	0.557	−8.77

## Data Availability

Not applicable.
